# The New York Register of Medicine and Pharmacy

**Published:** 1850-10

**Authors:** 


*The New York Register of Medicine and Pharmacy.* Edited by C. D. Griswold, M. D.

We have received the first number of a new Medical Journal, of the above title. It is published in the city of New York, in semi-monthly numbers, of sixteen pages, at one dollar per annum. As a laborer in the advancement of medical science, and sound practical knowledge, we greet this new candidate cordially, and wish it an abundant success. B.

